# Molecular epidemiology and antimicrobial resistance features of *Acinetobacter baumannii* clinical isolates from Pakistan

**DOI:** 10.1186/s12941-019-0344-7

**Published:** 2020-01-15

**Authors:** Nabil Karah, Fizza Khalid, Sun Nyunt Wai, Bernt Eric Uhlin, Irfan Ahmad

**Affiliations:** 1 The Laboratory for Molecular Infection Medicine Sweden, Department of Molecular Biology, and Umea Centre for Microbial Research, Umea University, Umeå, Sweden; 2 Department of Microbiology, University of Health Sciences, Lahore, Pakistan; 3 Institute of Biomedical and Allied Health Sciences, University of Health Sciences, Lahore, Pakistan

**Keywords:** Carbapenem-resistance, Strain typing, International clone, Phylogeny

## Abstract

**Background:**

*Acinetobacter baumannii* is a Gram-negative opportunistic pathogen with a notorious reputation of being resistant to antimicrobial agents. The capability of *A. baumannii* to persist and disseminate between healthcare settings has raised a major concern worldwide.

**Methods:**

Our study investigated the antibiotic resistance features and molecular epidemiology of 52 clinical isolates of *A. baumannii* collected in Pakistan between 2013 and 2015. Antimicrobial susceptibility patterns were determined by the agar disc diffusion method. Comparative sequence analyses of the *ampC* and *bla*_OXA-51-like_ alleles were used to assign the isolates into clusters. The whole genomes of 25 representative isolates were sequenced using the MiSeq Desktop Sequencer. Free online applications were used to determine the phylogeny of genomic sequences, retrieve the multilocus sequence types (ST), and detect acquired antimicrobial resistance genes.

**Results:**

Overall, the isolates were grouped into 7 clusters and 3 sporadic isolates. The largest cluster, Ab-Pak-cluster-1 (*bla*_OXA-66_ and IS*Aba1*-*ampC*-*19*) included 24 isolates, belonged to ST2 and International clone (IC) II, and was distributed between two geographical far-off cities, Lahore and Peshawar. Ab-Pak-clusters-2 (*bla*_OXA-66_, IS*Aba1*-*ampC*-*2*), and -3 (*bla*_OXA-66_, IS*Aba1*-*ampC*-*20*) and the individual isolate Ab-Pak-Lah-01 (IS*Aba1*-*bla*_OXA-66_, IS*Aba1*-*ampC*-*2*) were also assigned to ST2 and IC II. On the other hand, Ab-Pak-clusters-4 (*bla*_OXA-69_, *ampC*-*1*), -5 (*bla*_OXA-69_, IS*Aba1*-*ampC*-*78*), and -6A (*bla*_OXA-371_, IS*Aba1*-*ampC*-*3*) belonged to ST1, while Ab-Pak-cluster-6B (*bla*_OXA-371_, IS*Aba1*-*ampC*-*8*) belonged to ST1106, with both ST1 and ST1106 being members of IC I. Five isolates belonged to Ab-Pak-cluster-7 (*bla*_OXA-65_, *ampC*-*43*). This cluster corresponded to ST158, showed a well-delineated position on the genomic phylogenetic tree, and was equipped with several antimicrobial resistance genes including *bla*_OXA-23_ and *bla*_GES-11_.

**Conclusions:**

Our study detected the occurrence of 7 clusters of *A. baumannii* in Pakistan. Altogether, 6/7 of the clusters and 45/52 (86.5%) of the isolates belonged to IC I (*n *= 9) or II (*n *= 36), making Pakistan no exception to the global domination of these two clones. The onset of ST158 in Pakistan marked a geographical dispersal of this clone beyond the Middle East and brought up the need for a detailed characterization.

## Introduction

*Acinetobacter baumannii* is an important opportunistic pathogen that has increasingly been reported worldwide. The World Health Organization (WHO) has recently classified carbapenem resistant *A. baumannii* as a top priority organism for research and development of new antibiotics against antibiotic-resistant bacteria [1]. The occurrence of *A. baumannii* in Pakistan was first reported in 2004, where it counted for 4.4% of the Gram-negative bacilli in a collection of 812 isolates obtained from a variety of clinical samples in Rawalpindi in 2002 [2]. Importantly, 72% of the reported *A. baumannii* isolates were found to be extended-spectrum beta-lactamase (ESBL) producers. Later, eight clusters of carbapenem-resistant isolates of *A. baumannii* were detected in two intensive care units in Karachi, Pakistan, between November 2007 and August 2008 [3]. Concurrently, *A. baumannii* was the most frequent bacterial pathogen among a collection of 50 carbapenem-resistant clinical isolates obtained in Rawalpindi in 2009 [4]. According to Kaleem et al., twenty-seven (84%) of the carbapenem-resistant *A. baumannii* isolates were metallo-beta-lactamase positive by the E-test strip method.

Recently, *A. baumannii* was isolated in 18% of patients with ventilator-associated pneumonia, making it the 4th most frequent pathogen, at the Khyber Teaching Hospital in Peshawar in 2013 [5]. Notably, *A. baumannii* was declared as the most resistant pathogen identified in this study. *A. baumannii* also ranked the second most commonly detected pathogen (24.7%) among a collection of carbapenemase producers obtained from one hospital in Lahore between 2015 and 2016 [6]. Furthermore, *A. baumannii* was the main isolated bacterium (39.8%) among a total of 113 isolates recovered from 80 ventilator-supported patients at the Foundation Hospital, Rawalpindi, in 2016 [7].

Nevertheless, only few of these studies have plotted the epidemiology of *A. baumannii* in Pakistan onto a global map. Most of the 2007–2008 hospital-acquired carbapenem-resistant *A. baumannii* belonged to either International clone (IC) I or II [3]. Similarly, 1/17 and 7/17 clinical isolates of *A. baumannii*, obtained in 2008, were linked to ICs I and II, respectively [8]. Two isolates collected in Norway in 2009 with a history of import from Pakistan were assigned to sequence types 2 (ST2) and ST15, using the multilocus sequence typing (MLST) scheme of Pasteur Institute [9, 10]. ST2 is a key member of IC II while ST15 has often been reported in South America [11, 12]. Our search in the *A. baumannii* MLST databases yielded records of 4 isolates from Pakistan (last accessed in 11 September 2019). The 4 isolates were entered in 2019, which might explain why we could not find published literature on them. Two of these isolates belonged to ST1310 (8, 1, 5, 1, 6, 2, 3) or ST1327 (13, 1, 5, 26, 7, 1, 29), for which there was no linkage to known ICs. The other two isolates belonged to ST1106 (2, 1, 2, 1, 5, 1, 1), a member of clonal complex CC1 corresponding to IC I [11].

The aim of this study was to investigate the molecular epidemiology and antibiotic resistance features of 52 *A. baumannii* clinical isolates collected in Pakistan between 2013 and 2015. The clonality among our isolates was crossed over the global population of *A. baumannii*.

## Materials and methods

### Bacterial isolates

Two collections of *A. baumannii* clinical isolates were included in this study. The isolates, each recovered from one patient, were collected on a consecutive basis regardless of their antimicrobial susceptibility features (Table 1). The first collection (*n *= 16) was obtained between February and September 2013 at the Combined Military Hospital in Lahore (CMH Lahore), while the second one (*n *= 36) was obtained between February and November 2015 at the Combined Military Hospital in Peshawar (CMH Peshawar). The geographic distance between the two hospitals is about 520 kilometres (Additional file 1: Figure S1). The isolates were cultured from pus (*n *= 17), respiratory secretions (*n *= 27), intravenous catheter tip (*n *= 5), Foley catheter tip (*n *= 2), and bile (*n *= 1). The clinical samples were processed according to standard in-house culture protocols. When needed, isolates were revived from frozen glycerol stocks onto Brain Heart Infusion agar plates or slants (Oxoid, Basingstoke, United Kingdom). Species identification was first determined using API 20 NE (BioMerieux, France) and later confirmed by detecting occurrence of the intrinsic *bla*_OXA-51-like_ gene in all the isolates [13]. The identification was further verified by partial *rpoB* (zone 1, 352 bp) gene sequence analysis in 25 isolates selected for whole-genome sequencing [14].

**Table 1:** Epidemiological data and antimicrobial resistance patterns of the isolates

Sample ID	Specimen	Month and year of isolation	Hospital/ward	Antimicrobial susceptibility pattern	Antimicrobial resistance pattern
Ab-Pak-Lah-01	Tracheal secretions	Feb-2013	CMH Lahore/ICU	CST, PMB	SAM, TZP, FEP, CTX, CAZ, CRO, IPM, MEM, GM, AMK, TOB, CIP, LVX, SXT, MIN, DOX
Ab-Pak-Lah-02	Intravenous catheter tip	Feb-2013	CMH Lahore/ICU	CST, PMB, DOX	SAM, TZP, FEP, CTX, CAZ, CRO, IPM, MEM, GM, AMK, TOB, CIP, LVX, SXT, MIN
Ab-Pak-Lah-03	Bronchial lavage	Apr-2013	CMH Lahore/ICU	CST, PMB, DOX	SAM, TZP, FEP, CTX, CAZ, CRO, IPM, MEM, GM, AMK, TOB, CIP, LVX, SXT, MIN
Ab-Pak-Lah-04	Tracheal secretions	May-2013	CMH Lahore/ICU	CST, PMB, DOX	SAM, TZP, FEP, CTX, CAZ, CRO, IPM, MEM, GM, AMK, TOB, CIP, LVX, SXT, MIN
Ab-Pak-Lah-06	Bronchial lavage	May-2013	CMH Lahore/ICU	CST, PMB, DOX	SAM, TZP, FEP, CTX, CAZ, CRO, IPM, MEM, GM, AMK, TOB, CIP, LVX, SXT, MIN
Ab-Pak-Lah-07	Tracheal secretions	May-2013	CMH Lahore/ICU	CST, PMB, DOX	SAM, TZP, FEP, CTX, CAZ, CRO, IPM, MEM, GM, AMK, TOB, CIP, LVX, SXT, MIN
Ab-Pak-Lah-08	Intravenous catheter tip	Jun-2013	CMH Lahore/ICU	CST, PMB, DOX	SAM, TZP, FEP, CTX, CAZ, CRO, IPM, MEM, GM, AMK, TOB, CIP, LVX, SXT, MIN
Ab-Pak-Lah-09	Pus	Jun-2013	CMH Lahore/ICU	CST, PMB, DOX	SAM, TZP, FEP, CTX, CAZ, CRO, IPM, MEM, GM, AMK, TOB, CIP, LVX, SXT, MIN
Ab-Pak-Lah-10	Pus	Jul-2013	CMH Lahore/ICU	CST, PMB, DOX	SAM, TZP, FEP, CTX, CAZ, CRO, IPM, MEM, GM, AMK, TOB, CIP, LVX, SXT, MIN
Ab-Pak-Lah-11	Tracheal secretions	Jul-2013	CMH Lahore/ICU	CST, PMB, DOX	SAM, TZP, FEP, CTX, CAZ, CRO, IPM, MEM, GM, AMK, TOB, CIP, LVX, SXT, MIN
Ab-Pak-Lah-12	Pus	Aug-2013	CMH Lahore/ICU	CST, PMB, DOX	SAM, TZP, FEP, CTX, CAZ, CRO, IPM, MEM, GM, AMK, TOB, CIP, LVX, SXT, MIN
Ab-Pak-Lah-13	Tracheal secretions	Aug-2013	CMH Lahore/ICU	CST, PMB, DOX	SAM, TZP, FEP, CTX, CAZ, CRO, IPM, MEM, GM, AMK, TOB, CIP, LVX, SXT, MIN
Ab-Pak-Lah-14	Tracheal secretions	Sep-2013	CMH Lahore/ICU	CST, PMB, DOX	SAM, TZP, FEP, CTX, CAZ, CRO, IPM, MEM, GM, AMK, TOB, CIP, LVX, SXT, MIN
Ab-Pak-Lah-15	Tracheal secretions	Sep-2013	CMH Lahore/ICU	CST, PMB, DOX	SAM, TZP, FEP, CTX, CAZ, CRO, IPM, MEM, GM, AMK, TOB, CIP, LVX, SXT, MIN
Ab-Pak-Lah-16	Tracheal secretions	Sep-2013	CMH Lahore/ICU	CST, PMB, DOX	SAM, TZP, FEP, CTX, CAZ, CRO, IPM, MEM, GM, AMK, TOB, CIP, LVX, SXT, MIN
Ab-Pak-Lah-17	Tracheal secretions	Sep-2013	CMH Lahore/ICU	CST, PMB, DOX	SAM, TZP, FEP, CTX, CAZ, CRO, IPM, MEM, GM, AMK, TOB, CIP, LVX, SXT, MIN
Ab-Pak-Pesh-01	Nasobronchial lavage	Feb-2015	CMH Peshawar/ICU	CST, PMB, MIN, DOX	SAM, TZP, FEP, CTX, CAZ, CRO, IPM, MEM, GM, AMK, TOB, CIP, LVX, SXT
Ab-Pak-Pesh-02	Nasobronchial lavage	Feb-2015	CMH Peshawar/ICU	CST, PMB, MIN, DOX	SAM, TZP, FEP, CTX, CAZ, CRO, IPM, MEM, GM, AMK, TOB, CIP, LVX, SXT
Ab-Pak-Pesh-03	Pus	Feb-2015	CMH Peshawar/ICU	CST, PMB, MIN	SAM, TZP, FEP, CTX, CAZ, CRO, IPM, MEM, GM, AMK, TOB, CIP, LVX, SXT, DOX
Ab-Pak-Pesh-04	Intravenous catheter tip	Mar-2015	CMH Peshawar/MW	CST, PMB, MIN	SAM, TZP, FEP, CTX, CAZ, CRO, IPM, MEM, GM, AMK, TOB, CIP, LVX, SXT, DOX
Ab-Pak-Pesh-05	Nasobronchial lavage	Mar-2015	CMH Peshawar/ICU	CST, PMB, MIN, DOX, AMK	SAM, TZP, FEP, CTX, CAZ, CRO, IPM, MEM, GM, TOB, CIP, LVX, SXT
Ab-Pak-Pesh-06	Pus	Apr-2015	CMH Peshawar/ICU	CST, PMB, MIN	SAM, TZP, FEP, CTX, CAZ, CRO, IPM, MEM, GM, AMK, TOB, CIP, LVX, SXT, DOX
Ab-Pak-Pesh-07	Nasobronchial lavage	Apr-2015	CMH Peshawar/ICU	CST, PMB, MIN, DOX	SAM, TZP, FEP, CTX, CAZ, CRO, IPM, MEM, GM, AMK, TOB, CIP, LVX, SXT
Ab-Pak-Pesh-08	Endobronchial secretions	Apr-2015	CMH Peshawar/MW	CST, PMB, MIN	SAM, TZP, FEP, CTX, CAZ, CRO, IPM, MEM, GM, AMK, TOB, CIP, LVX, SXT, DOX
Ab-Pak-Pesh-09	Nasobronchial lavage	May-2015	CMH Peshawar/ICU	CST, PMB, MIN, DOX	SAM, TZP, FEP, CTX, CAZ, CRO, IPM, MEM, GM, AMK, TOB, CIP, LVX, SXT
Ab-Pak-Pesh-10	Intravenous catheter tip	May-2015	CMH Peshawar/ICU	CST, PMB, MIN, DOX	SAM, TZP, FEP, CTX, CAZ, CRO, IPM, MEM, GM, AMK, TOB, CIP, LVX, SXT
Ab-Pak-Pesh-11	Nasobronchial lavage	May-2015	CMH Peshawar/ICU	CST, PMB, MIN	SAM, TZP, FEP, CTX, CAZ, CRO, IPM, MEM, GM, AMK, TOB, CIP, LVX, SXT, DOX
Ab-Pak-Pesh-13	Nasobronchial lavage	May-2015	CMH Peshawar/ICU	CST, PMB, MIN	SAM, TZP, FEP, CTX, CAZ, CRO, IPM, MEM, GM, AMK, TOB, CIP, LVX, SXT, DOX
Ab-Pak-Pesh-14	Endobronchial secretions	May-2015	CMH Peshawar/MW	CST, PMB, MIN	SAM, TZP, FEP, CTX, CAZ, CRO, IPM, MEM, GM, AMK, TOB, CIP, LVX, SXT, DOX
Ab-Pak-Pesh-15	Pus	May-2015	CMH Peshawar/ICU	CST, PMB, MIN, DOX	SAM, TZP, FEP, CTX, CAZ, CRO, IPM, MEM, GM, AMK, TOB, CIP, LVX, SXT
Ab-Pak-Pesh-16	Pus	May-2015	CMH Peshawar/ICU	CST, PMB, MIN, DOX	SAM, TZP, FEP, CTX, CAZ, CRO, IPM, MEM, GM, AMK, TOB, CIP, LVX, SXT
Ab-Pak-Pesh-17	Nasobronchial lavage	May-2015	CMH Peshawar/ICU	CST, PMB, MIN, DOX	SAM, TZP, FEP, CTX, CAZ, CRO, IPM, MEM, GM, AMK, TOB, CIP, LVX, SXT
Ab-Pak-Pesh-18	Pus	Jun-2015	CMH Peshawar/MW	CST, PMB, MIN, DOX	SAM, TZP, FEP, CTX, CAZ, CRO, IPM, MEM, GM, AMK, TOB, CIP, LVX, SXT
Ab-Pak-Pesh-19	Pus	Jun-2015	CMH Peshawar/ICU	CST, PMB, MIN	SAM, TZP, FEP, CTX, CAZ, CRO, IPM, MEM, GM, AMK, TOB, CIP, LVX, SXT, DOX
Ab-Pak-Pesh-20	Pus	Jun-2015	CMH Peshawar/MW	CST, PMB, MIN, DOX	SAM, TZP, FEP, CTX, CAZ, CRO, IPM, MEM, GM, AMK, TOB, CIP, LVX, SXT
Ab-Pak-Pesh-21	Bile	Jun-2015	CMH Peshawar/MW	CST, PMB, MIN	SAM, TZP, FEP, CTX, CAZ, CRO, IPM, MEM, GM, AMK, TOB, CIP, LVX, SXT, DOX
Ab-Pak-Pesh-22	Pus	Jul-2015	CMH Peshawar/ICU	CST, PMB, MIN, DOX, IPM	SAM, TZP, FEP, CTX, CAZ, CRO, MEM, GM, AMK, TOB, CIP, LVX, SXT
Ab-Pak-Pesh-23	Pus	Jul-2015	CMH Peshawar/ICU	CST, PMB, MIN, DOX	SAM, TZP, FEP, CTX, CAZ, CRO, IPM, MEM, GM, AMK, TOB, CIP, LVX, SXT
Ab-Pak-Pesh-24	Foley catheter tip	Jul-2015	CMH Peshawar/MW	CST, PMB, MIN, DOX	SAM, TZP, FEP, CTX, CAZ, CRO, IPM, MEM, GM, AMK, TOB, CIP, LVX, SXT
Ab-Pak-Pesh-25	Pus	Jul-2015	CMH Peshawar/ICU	CST, PMB	SAM, TZP, FEP, CTX, CAZ, CRO, IPM, MEM, GM, AMK, TOB, CIP, LVX, SXT, DOX, MIN
Ab-Pak-Pesh-26	Nasobronchial lavage	Jul-2015	CMH Peshawar/ICU	CST, PMB, MIN, DOX	SAM, TZP, FEP, CTX, CAZ, CRO, IPM, MEM, GM, AMK, TOB, CIP, LVX, SXT
Ab-Pak-Pesh-27	Nasobronchial lavage	Aug-2015	CMH Peshawar/ICU	CST, PMB, MIN, DOX	SAM, TZP, FEP, CTX, CAZ, CRO, IPM, MEM, GM, AMK, TOB, CIP, LVX, SXT
Ab-Pak-Pesh-28	Pus	Aug-2015	CMH Peshawar/ICU	CST, PMB, MIN, DOX	SAM, TZP, FEP, CTX, CAZ, CRO, IPM, MEM, GM, AMK, TOB, CIP, LVX, SXT
Ab-Pak-Pesh-29	Pus	Aug-2015	CMH Peshawar/ICU	CST, PMB, MIN	SAM, TZP, FEP, CTX, CAZ, CRO, IPM, MEM, GM, AMK, TOB, CIP, LVX, SXT, DOX
Ab-Pak-Pesh-30	Pus	Aug-2015	CMH Peshawar/MW	CST, PMB, MIN, DOX	SAM, TZP, FEP, CTX, CAZ, CRO, IPM, MEM, GM, AMK, TOB, CIP, LVX, SXT
Ab-Pak-Pesh-31	Pus	Sep-2015	CMH Peshawar/ICU	CST, PMB, MIN	SAM, TZP, FEP, CTX, CAZ, CRO, IPM, MEM, GM, AMK, TOB, CIP, LVX, SXT, DOX
Ab-Pak-Pesh-32	Endobronchial secretions	Sep-2015	CMH Peshawar/MW	CST, PMB, MIN	SAM, TZP, FEP, CTX, CAZ, CRO, IPM, MEM, GM, AMK, TOB, CIP, LVX, SXT, DOX
Ab-Pak-Pesh-33	Endobronchial secretions	Sep-2015	CMH Peshawar/MW	CST, PMB, MIN, DOX	SAM, TZP, FEP, CTX, CAZ, CRO, IPM, MEM, GM, AMK, TOB, CIP, LVX, SXT
Ab-Pak-Pesh-34	Nasobronchial lavage	Sep-2015	CMH Peshawar/ICU	CST, PMB, MIN, DOX	SAM, TZP, FEP, CTX, CAZ, CRO, IPM, MEM, GM, AMK, TOB, CIP, LVX, SXT
Ab-Pak-Pesh-35	Foley catheter tip	Oct-2015	CMH Peshawar/MW	CST, PMB, MIN	SAM, TZP, FEP, CTX, CAZ, CRO, IPM, MEM, GM, AMK, TOB, CIP, LVX, SXT, DOX
Ab-Pak-Pesh-36	Intravenous catheter tip	Oct-2015	CMH Peshawar/MW	CST, PMB, MIN	SAM, TZP, FEP, CTX, CAZ, CRO, IPM, MEM, GM, AMK, TOB, CIP, LVX, SXT, DOX
Ab-Pak-Pesh-37	Nasobronchial lavage	Nov-2015	CMH Peshawar/ICU	CST, PMB, MIN, DOX	SAM, TZP, FEP, CTX, CAZ, CRO, IPM, MEM, GM, AMK, TOB, CIP, LVX, SXT

*CMH* combined military hospital, *ICU* intensive care unit, *MW* medical ward, *SAM* ampicillin/sulbactam, *TZP* piperacillin/tazobactam, *FEP* cefepime, *CTX* cefotaxime, *CAZ* ceftazidime, *CRO* ceftriaxone, *IPM* imipenem, *MEM* meropenem, *CST* colistin, *PMB* polymyxin B, *DOX* doxycycline, *MIN* minocycline, *GM* gentamicin, *AMK* amikacin, *TOB* tobramycin, *CIP* ciprofloxacin, *LVX* levofloxacin, *SXT* trimethoprim/sulfamethoxazole

### Antimicrobial susceptibility testing

Susceptibility of the isolates to ampicillin/sulbactam, piperacillin/tazobactam, cefepime, cefotaxime, ceftazidime, ceftriaxone, meropenem, imipenem, gentamicin, amikacin, tobramycin, ciprofloxacin, levofloxacin, trimethoprim/sulfamethoxazole, and minocycline was investigated by the agar disc diffusion method, using discs from Oxoid (Basingstoke, United Kingdom). The broth microdilution method was used for doxycycline (Pfizer Global pharmaceuticals), polymyxin B (Glaxosmith Kline pharmaceuticals), and colistin (Forest pharmaceuticals). The tests were performed and susceptibility patterns were interpreted following the guidelines of the Clinical and Laboratory Standards Institute (CLSI) [15].

### Strain typing

The isolates were typed using two single-locus molecular schemes based on the allelic identity of the *A. baumannii*-intrinsic *ampC* and *bla*_OXA-51-like_ genes [16, 17]. PCR amplifications of *ampC* and *bla*_OXA-51-like_ were performed using in-house designed primers (Additional file 2: Table S1) and followed by Sanger sequencing of the amplicons, as previously described [16, 17]. This approach was able to detect the occurrence of insertion sequence (IS) elements, such as IS*Aba1*, in the bordering regions of *ampC* and/or *bla*_OXA-51-like_.

### Whole-genome sequence analyses

Twenty-five isolates were selected for whole-genome sequencing based on their antimicrobial resistance patterns and strain typing results. Sequencing was performed using the MiSeq Desktop Sequencer and MiSeq Reagent Kit v3 (Illumina, San Diego, CA, USA). DNA preparation, library construction, and genome sequencing were done according to the manufacturers’ instructions. Sequence data were assembled and analysed using the CLC genomics workbench (v7.0.4; CLC bio, Aarhus, Denmark). The web server Reference sequence Alignment-based Phylogeny builder (REALPHY) was used to construct a phylogenetic tree based on multiple alignments of the genomic sequence data [18]. Sequence reads were mapped to the genomes of three well-known reference strains: AYE (IC I, GenBank: NC_010410.1), ACICU (IC II, GenBank: NC_010611.1), and ATCC 17978 (sporadic ST437, GenBank: NZ_CP018664.1).

An *In*-*silico* search was performed to detect the existence of 24 *A. baumannii* plasmid-borne replicase genes, according to the *A. baumannii* PCR-based replicon typing (AB-PBRT) scheme and a number of other studies on *A. baumannii* plasmids [19–22]. The MLST web-based search engine, hosted by the Center for Genomic Epidemiology in Denmark (http://www.genomicepidemiology.org/), was used to assign the isolates into STs according to the Institute Pasteur’s MLST scheme (http://www.pasteur.fr/mlst) [23]. The occurrence of acquired antimicrobial resistance genes was detected using the ResFinder service, also hosted by the Center for Genomic Epidemiology in Denmark [24]. The occurrence of resistance genes was verified, and genetic surroundings were annotated based on the yields of nucleotide similarities obtained using the Basic Local Alignment Search Tool (http://blast.ncbi.nlm.nih.gov/Blast.cgi) against the “Nucleotide collection (nr/nt)” and/or “Whole-genome shotgun contigs (wgs)” databases [25]. The presence of neighbouring IS elements was detected using the ISfinder online application [26].

### Nucleotide sequence accession numbers

Draft genome sequences of all the isolates were deposited in the DDBJ/EMBL/GenBank database under the BioProject accession number: PRJNA482499. The versions described in this paper are QQPR01000000, SMUA01000000, QQPS01000000, QQPT01000000, SMUB01000000, SMUC01000000, SMUD01000000, QQPU01000000, QQPV01000000, QQPW01000000, SMUE01000000, SMUF01000000, SMUG01000000, SMUQ01000000, QQPX01000000, SMUR01000000, QQPY01000000, SMUH01000000, SMUI01000000, QQPZ01000000, SMUJ01000000, QQQA01000000, QQQB01000000, SMUK01000000, and QQQC01000000. The new alleles *ampC*-*78* and *ampC*-*79* were deposited in the *ampC* database hosted at the *A. baumannii* MLST website (http://pubmlst.org/abaumannii/) and in the DDBJ/EMBL/GenBank database under the accession numbers MK634301.1 (*bla*_ADC-191_) and MK690541.1 (*bla*_ADC-91_), respectively.

## Results and discussion

### Antimicrobial resistance patterns

The isolates showed resistance rates of 100% (52/52) to ampicillin/sulbactam, piperacillin/tazobactam, cefepime, cefotaxime, ceftazidime, ceftriaxone, meropenem, gentamicin, tobramycin, ciprofloxacin, levofloxacin, and trimethoprim/sulfamethoxazole (Table 1 and Additional file 3: Table S2). In addition, 98.1% (51/52) of the isolates were resistant to amikacin and imipenem, 32.7% (17/52) to minocycline, 30.8% (16/52) to doxycycline, and 0% (0/52) to colistin and polymyxin B (Table 1 and Additional file 3: Table S2). All the isolates were resistant to ≥ 3 classes of antibiotics and were accordingly defined as multidrug-resistant [27]. The results were in line with previous studies describing extensive occurrence of multidrug-resistant strains of *A. baumannii* in Pakistan [28–30]. However, our results could be partially biased by the predominance of few genetically related clusters of isolates, as described in the next paragraph.

### Strain typing

Comparative sequence analysis of the *bla*_OXA-51-like_ and *ampC* loci enabled us to group 49/52 of the isolates into seven clusters, designated Ab-Pak-cluster-1 to -7 (Table 2). The remaining 3 isolates (Ab-Pak-Lah-01, Ab-Pak-Pesh-04 and Ab-Pak-Pesh-22) were considered as individual strains. Ab-Pak-cluster-1 (*bla*_OXA-66_, IS*Aba1*-*ampC*-*19*) was the largest cluster with 24 isolates distributing between Lahore, 2013 (*n *= 14) and Peshawar, 2015 (*n *= 10). The second largest cluster, Ab-Pak-cluster-2 (*bla*_OXA-66_, IS*Aba1*-*ampC*-*2*), included 7 isolates, which were all detected in Peshawar, 2015. Ab-Pak-cluster-3 (*bla*_OXA-66_, IS*Aba1*-*ampC*-*20*) included 4 isolates and was also exclusive to Peshawar, 2015. Individual strain Ab-Pak-Lah-01 (IS*Aba1*-*bla*_OXA-66_, IS*Aba1*-*ampC*-*2)* was the only isolate in our collection carrying insertion sequence IS*Aba1* upstream its intrinsic *bla*_OXA-51-like_ gene (Table 2). Based on their *bla*_OXA-51-like_ and *ampC* alleles, Ab-Pak-clusters-1, -2 and -3, and individual strain Ab-Pak-Lah-01 belonged to *A. baumannii* IC II [11, 16, 17].

**Table 2:** Molecular strain typing of the isolates

Sample ID	*bla*_OXA-51-like_ allele	*ampC* allele	*bla*_OXA-51-like_- and *ampC*-based clusterization	Whole genome sequencing	Multilocus sequence typing		
					ST profile^b^	ST	CC
Ab-Pak-Lah-01	IS*Aba1, bla*_OXA-66_	IS*Aba1*, *ampC*-*2*	Individual strain	QQPR00000000.1	2, 2, 2, 2, 2, 2, 2	2	2
Ab-Pak-Lah-02	*bla*_OXA-66_	IS*Aba1, ampC*-*19*	Ab-Pak-cluster-1	ND	–	–	
Ab-Pak-Lah-03	*bla*_OXA-66_	IS*Aba1, ampC*-*19*	Ab-Pak-cluster-1	ND	–	–	
Ab-Pak-Lah-04	*bla*_OXA-66_	IS*Aba1, ampC*-*19*	Ab-Pak-cluster-1	SMUA00000000.1	2, 2, 2, 2, 2, 2, 2	2	2
Ab-Pak-Lah-06	*bla*_OXA-66_	IS*Aba1, ampC*-*19*	Ab-Pak-cluster-1	ND	–	–	
Ab-Pak-Lah-07	*bla*_OXA-66_	IS*Aba1, ampC*-*19*	Ab-Pak-cluster-1	ND	–	–	
Ab-Pak-Lah-08	*bla*_OXA-66_	IS*Aba1, ampC*-*19*	Ab-Pak-cluster-1	QQPS00000000.1	2, 2, 2, 2, 2, 2, 2	2	2
Ab-Pak-Lah-09	*bla*_OXA-66_	IS*Aba1, ampC*-*19*	Ab-Pak-cluster-1	ND	–	–	
Ab-Pak-Lah-10	*bla*_OXA-66_	IS*Aba1, ampC*-*19*	Ab-Pak-cluster-1	ND	–	–	
Ab-Pak-Lah-11	*bla*_OXA-66_	IS*Aba1, ampC*-*19*	Ab-Pak-cluster-1	ND	–	–	
Ab-Pak-Lah-12	*bla*_OXA-66_	IS*Aba1, ampC*-*19*	Ab-Pak-cluster-1	ND	–	–	
Ab-Pak-Lah-13	*bla*_OXA-66_	IS*Aba1, ampC*-*19*	Ab-Pak-cluster-1	ND	–	–	
Ab-Pak-Lah-14	*bla*_OXA-371_	IS*Aba1, ampC*-*3*	Ab-Pak-cluster-6A	QQPT00000000.1	1, 1, 1, 1, 5, 1, 1	1	1
Ab-Pak-Lah-15	*bla*_OXA-66_	IS*Aba1, ampC*-*19*	Ab-Pak-cluster-1	ND	–	–	
Ab-Pak-Lah-16	*bla*_OXA-66_	IS*Aba1, ampC*-*19*	Ab-Pak-cluster-1	ND	–	–	
Ab-Pak-Lah-17	*bla*_OXA-66_	IS*Aba1, ampC*-*19*	Ab-Pak-cluster-1	ND	–	–	
Ab-Pak-Pesh-01	*bla*_OXA-65_^a^	*ampC*-*43*	Ab-Pak-cluster-7	SMUB00000000.1	41, 42, 13, 1, 5, 4, 14	158	158
Ab-Pak-Pesh-02	*bla*_OXA-65_^a^	*ampC*-*43*	Ab-Pak-cluster-7	ND	–	–	
Ab-Pak-Pesh-03	*bla*_OXA-66_	IS*Aba1*, *ampC*-*2*	Ab-Pak-cluster-2	ND	–	–	
Ab-Pak-Pesh-04	*bla*_OXA-64_	*ampC*-*25*	Individual strain	SMUC00000000.1	3, 3, 2, 4, 7, 2, 4	25	25
Ab-Pak-Pesh-05	*bla*_OXA-371_	IS*Aba1, ampC*-*8*	Ab-Pak-cluster-6B	SMUD00000000.1	2, 1, 2, 1, 5, 1, 1	1106	1
Ab-Pak-Pesh-06	*bla*_OXA-66_	IS*Aba1*, *ampC*-*2*	Ab-Pak-cluster-2	QQPU00000000.1	2, 2, 2, 2, 2, 2, 2	2	2
Ab-Pak-Pesh-07	*bla*_OXA-65_^a^	*ampC*-*43*	Ab-Pak-cluster-7	QQPV00000000.1	41, 42, 13, 1, 5, 4, 14	158	158
Ab-Pak-Pesh-08	*bla*_OXA-66_	IS*Aba1*, *ampC*-*2*	Ab-Pak-cluster-2	ND	–	–	
Ab-Pak-Pesh-09	*bla*_OXA-65_^a^	*ampC*-*43*	Ab-Pak-cluster-7	ND	–	–	
Ab-Pak-Pesh-10	*bla*_OXA-66_	IS*Aba1, ampC*-*19*	Ab-Pak-cluster-1	QQPW00000000.1	2, 2, 2, 2, 2, 2, 2	2	2
Ab-Pak-Pesh-11	*bla*_OXA-66_	IS*Aba1*, *ampC*-*2*	Ab-Pak-cluster-2	SMUE00000000.1	2, 2, 2, 2, 2, 2, 2	2	2
Ab-Pak-Pesh-13	*bla*_OXA-66_	IS*Aba1*, *ampC*-*2*	Ab-Pak-cluster-2	SMUF00000000.1	2, 2, 2, 2, 2, 2, 2	2	2
Ab-Pak-Pesh-14	*bla*_OXA-69_	IS*Aba1, ampC*-*78*	Ab-Pak-cluster-5	SMUG00000000.1	1, 1, 1, 1, 5, 1, 1	1	1
Ab-Pak-Pesh-15	*bla*_OXA-66_	IS*Aba1, ampC*-*19*	Ab-Pak-cluster-1	ND	–	–	
Ab-Pak-Pesh-16	*bla*_OXA-66_	IS*Aba1, ampC*-*19*	Ab-Pak-cluster-1	SMUQ00000000.1	2, 2, 2, 2, 2, 2, 2	2	2
Ab-Pak-Pesh-17	*bla*_OXA-66_	IS*Aba1, ampC*-*19*	Ab-Pak-cluster-1	ND	–	–	
Ab-Pak-Pesh-18	*bla*_OXA-66_	IS*Aba1, ampC*-*19*	Ab-Pak-cluster-1	ND	–	–	
Ab-Pak-Pesh-19	*bla*_OXA-66_	IS*Aba1*, *ampC*-*2*	Ab-Pak-cluster-2	ND	–	–	
Ab-Pak-Pesh-20	*bla*_OXA-69_	*ampC*-*1*	Ab-Pak-cluster-4	QQPX00000000.1	1, 1, 1, 1, 5, 1, 1	1	1
Ab-Pak-Pesh-21	*bla*_OXA-66_	IS*Aba1*, *ampC*-*20*	Ab-Pak-cluster-3	ND	–	–	
Ab-Pak-Pesh-22	*bla*_OXA-68_	IS*Aba1, ampC*-*79*	Individual strain	SMUR00000000.1	1, 3, 10, 1, 4, 4, 4	23	23
Ab-Pak-Pesh-23	*bla*_OXA-66_	IS*Aba1, ampC*-*19*	Ab-Pak-cluster-1	ND	–	–	
Ab-Pak-Pesh-24	*bla*_OXA-66_	IS*Aba1, ampC*-*19*	Ab-Pak-cluster-1	ND	–	–	
Ab-Pak-Pesh-25	*bla*_OXA-66_	IS*Aba1, ampC*-*19*	Ab-Pak-cluster-1	QQPY00000000.1	2, 2, 2, 2, 2, 2, 2	2	2
Ab-Pak-Pesh-26	*bla*_OXA-371_	IS*Aba1, ampC*-*3*	Ab-Pak-cluster-6A	SMUH00000000.1	1, 1, 1, 1, 5, 1, 1	1	1
Ab-Pak-Pesh-27	*bla*_OXA-69_	*ampC*-*1*	Ab-Pak-cluster-4	SMUI00000000.1	1, 1, 1, 1, 5, 1, 1	1	1
Ab-Pak-Pesh-28	*bla*_OXA-65_^a^	*ampC*-*43*	Ab-Pak-cluster-7	QQPZ00000000.1	41, 42, 13, 1, 5, 4, 14	158	158
Ab-Pak-Pesh-29	*bla*_OXA-69_	IS*Aba1*, *ampC*-*78*	Ab-Pak-cluster-5	SMUJ00000000.1	1, 1, 1, 1, 5, 1, 1	1	1
Ab-Pak-Pesh-30	*bla*_OXA-69_	*ampC*-*1*	Ab-Pak-cluster-4	ND	–	–	
Ab-Pak-Pesh-31	*bla*_OXA-66_	IS*Aba1*, *ampC*-*20*	Ab-Pak-cluster-3	QQQA00000000.1	2, 2, 2, 2, 2, 2, 2	2	2
Ab-Pak-Pesh-32	*bla*_OXA-66_	IS*Aba1*, *ampC*-*20*	Ab-Pak-cluster-3	ND	–	–	
Ab-Pak-Pesh-33	*bla*_OXA-66_	IS*Aba1, ampC*-*19*	Ab-Pak-cluster-1	QQQB00000000.1	2, 2, 2, 2, 2, 2, 2	2	2
Ab-Pak-Pesh-34	*bla*_OXA-66_	IS*Aba1, ampC*-*19*	Ab-Pak-cluster-1	ND	–	–	
Ab-Pak-Pesh-35	*bla*_OXA-66_	IS*Aba1*, *ampC*-*2*	Ab-Pak-cluster-2	ND	–	–	
Ab-Pak-Pesh-36	*bla*_OXA-66_	IS*Aba1*, *ampC*-*20*	Ab-Pak-cluster-3	SMUK00000000.1	2, 2, 2, 2, 2, 2, 2	2	2
Ab-Pak-Pesh-37	*bla*_OXA-371_	IS*Aba1*, *ampC*-*8*	Ab-Pak-cluster-6B	QQQC00000000.1	2, 1, 2, 1, 5, 1, 1	1106	1

*ND* not determined, *ST* sequence type, *CC* clonal complex

^a^The nucleotide sequence of *bla*_OXA-65_ in our isolates had 3 synonymous nucleotide substitutions in comparison to the first GenBank-deposited allele for OXA-65 (GenBank: NG_049805.1)

^b^The ST profiles consist of 7 allele numbers corresponding to *cpn60*, *fusA*, *gltA*, *pyrG*, *recA*, *rplB*, and *rpoB*, respectively (https://pubmlst.org/abaumannii/)

Ab-Pak-cluster-4 (*bla*_OXA-69_, *ampC*-*1*) and Ab-Pak-cluster-5 (*bla*_OXA-69_, IS*Aba1*-*ampC*-78) included 3 and 2 isolates, respectively, and were both collected in Peshawar, 2015. The *ampC*-*78* allele had a novel sequence according to the nucleotide database of *ampC* alleles in *A. baumannii* (https://pubmlst.org/abaumannii/). Ab-Pak-cluster-6 (*bla*_OXA-371_) included 4 isolates scattering between Lahore, 2013 (1 isolate) and Peshawar, 2015 (3 isolates). The 4 isolates were equally divided into two sub-clusters, namely Ab-Pak-sub-cluster-6A (*bla*_OXA-371_, IS*Aba1*-*ampC*-*3*) and -6B (*bla*_OXA-371_, IS*Aba1*-*ampC*-*8*). The nucleotide identity of *ampC*-*8* (1167 bp) is 100% identical to *ampC*-*3* (1152 bp) apart from having a duplicated segment of 15 nucleotides (Additional file 4: File S1). The *bla*_OXA-371_ allele was only one nucleotide different in comparison to *bla*_OXA-69_ (GenBank accession numbers NG_049662.1 and NG_049809.1). Ab-Pak-clusters-4, -5, -6A, and -6B could conceivably be classified under the umbrella of IC I [16, 17, 31].

Ab-Pak-cluster-7 (*bla*_OXA-65_, *ampC*-*43*) included 5 isolates that were collected in Peshawar, 2015. The *bla*_OXA-65_ allele in Ab-Pak-cluster-7 encoded for ß-lactamase OXA-65 (GenBank: WP_001021782.1). However, this allele had 3 synonymous nucleotide substitutions in comparison to the first GenBank-deposited allele for OXA-65 (GenBank: NG_049805.1). This deviation revealed an inherent shortage in the current numbering system of the *bla*_OXA_ genes [32, 33]. In this regard, we would restate our view that alleles of well-defined bacterial genes should be numbered based on their nucleotide identifies rather than their amino acid sequences [16]. Lastly, the individual strains Ab-Pak-Pesh-04 (*bla*_OXA-64_, *ampC*-*25*) and Ab-Pak-Pesh-22 (*bla*_OXA-68_, IS*Aba1*-*ampC*-*79*) were assigned into CC25 and CC23, respectively [34, 35].

### Whole-genome phylogenetic tree

**Fig. 1:**
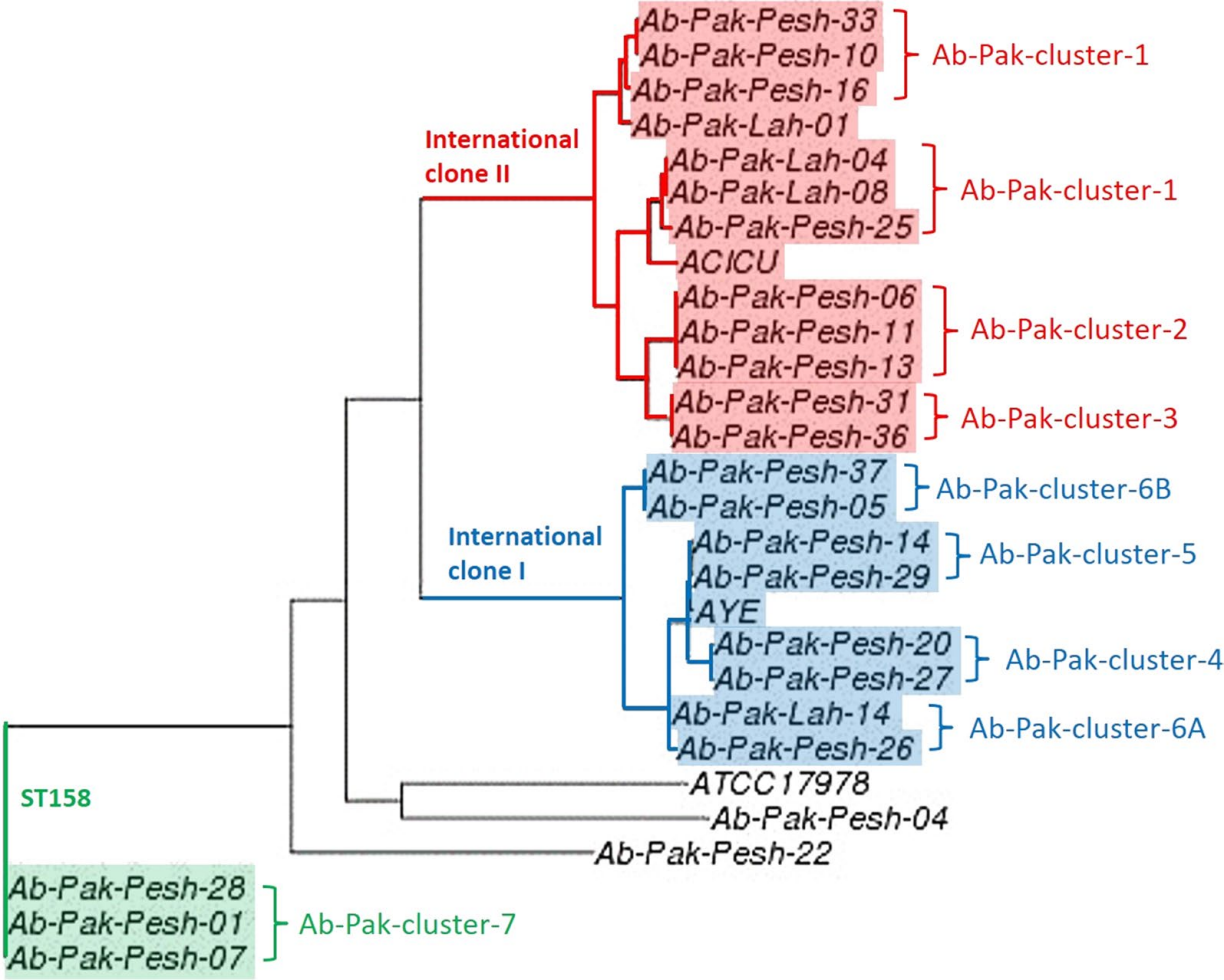
Phylogeny of the genomic sequence data of 25 representative strains. The phylogenetic tree was generated based on multiple alignments of the whole-genome sequences using REALPHY (https://realphy.unibas.ch/realphy/). Genomes of *A. baumannii* strains AYE, ACICU, and ATCC 17978 were included as reference strains of International clone (IC) I, IC II, and the sporadic sequence type ST437, respectively (GenBank accession numbers: NC_010410.1, NC_010611.1, and NZ_CP018664.1, respectively). Clusters, based on comparative sequence analysis of the *ampC* and *bla*_OXA-51-like_ loci, were indicated in text boxes. Branches, isolates and clusters belonging to ICs I and II were colored in red and blue, respectively. Branches, isolates and cluster belonging to ST158 were colored in green

Among the 25/52 isolates selected for whole genome sequence analysis, Ab-Pak-clusters-1, -2, -3, -4, -5, -6A, -6B, -7 were represented by 6 (showing 3 different antimicrobial resistance patterns)/24, 3/7, 2/4, 2/3, 2/2, 2/2, 2/2, and 3/5 isolates, respectively. The three individual isolates were also included. The genome assembly features were presented in Additional file 5: Table S3. The whole-genome phylogenetic tree showed a branching pattern indorsing the assembly of Ab-Pak-cludters-2, -3, -4, -5 and -7 (Fig. 1). The distinction between Ab-Pak-cludters-6A and -6B was also confirmed. The six isolates of Ab-Pak-cluster-1 were distributed on two detached branches and a few sub-branches. Such a high discriminatory power of whole-genome phylogenetic analyses, compared to loci-focused strain typing approaches, has been noted in previous studies [36, 37]. Nevertheless, the internal splits in Ab-Pak-cluster-1 were most likely related to a long-standing presence of this cluster. In addition, the tree showed a noticeably well-demarcated positioning of Ab-Pak-cluster-7 far from ICs I and II. Sequencing the whole genome of more isolates, especially from Ab-Pak-cluster-1, would have increased our capacity to confirm or exclude transmission events between patients [38]. However, such a limitation is generally tolerable when resources are limited.

### Plasmid replicase genes

The isolates carried between 1 and 4 plasmid replicase genes (Additional file 6: Table S4). The most commonly detected genes were *repAci6* (16/25) and *repAci1*/*repAci2* (15/25), which was in line with earlier studies elsewhere [39]. Interestingly, *repAci9* was detected in 12/25 of the isolates. The *repAci9*-positive plasmids ranged in size between 10,000 and 16,000 base pairs (bp), similarly to what has commonly been described (GenBank accession numbers: LT594096.1, CP024577.1, CP040088.1, CP035673.1, and AY541809.1). However, the individual isolate Ab-Pak-Pesh-22 carried a relatively large *repAci9*-positive plasmid, with the size of 31,442 bp (GenBank: SMUR01000022.1), on which the *tet(39)* tetracycline resistance operon was detected.

Other replicase genes, such as *repAci4* and the replicase genes of p2ABSDF and p4AYE, were present in 2/25, 5/25, and 4/25 of the isolates, respectively. Two isolates, Ab-Pak-Pesh-20 and Ab-Pak-Pesh-27, from Ab-Pak-cluster-4 carried a replicase gene with novel nucleotide identity, designated *repAci24*, showing only 58% nucleotide identity to the replicase gene of p3ABSDF (GenBank: CU468233.1). The three Ab-Pak-cluster-7 isolates carried another novel replicase gene, designated *repAci25*, which will be further discussed below. The occurrence of partial sequences of some replicase genes was noted in the genomes of few isolates (Additional file 6: Table S4). Our in silico analysis was not able to confirm or exclude if these incomplete sequences represented or belonged to actual plasmids.

### Multilocus sequence types

The selected representatives from Ab-Pak-clusters-1, -2, and -3 and individual strain Ab-Pak-Lah-01 were all assigned to ST2 (2, 2, 2, 2, 2, 2, 2), as shown in Table 2. Ab-Pak-clusters-4, -5, and -6A belonged to ST1 (1, 1, 1, 1, 5, 1, 1), whereas Ab-Pak-cluster-6B belonged to ST1106 (2, 1, 2, 1, 5, 1, 1). ST1 and ST1106 shared identical alleles in 5/7 of the loci. Ab-Pak-cluster-7 was assigned to ST158 (41, 42, 13, 1, 5, 4, 14). The individual isolates Ab-Pak-Pesh-04 and Ab-Pak-Pesh-22 belonged to ST25 (3, 3, 2, 4, 7, 2, 4) and ST23 (1, 3, 10, 1, 4, 4, 4), respectively. Overall, the MLST results were consistent with the two single-loci, *bla*_OXA-51-like_ and *ampC*, typing results and with the whole-genome-based phylogenetic analysis.

### Ab-Pak-cluster-7, a new member of clone ST158

Searching for homologous sequences in the GenBank databases enabled us to detect two *A. baumannii* strains, K50 (GenBank: OHJL00000000.1) and AA-014 (GenBank: AMGA00000000.1), carrying identical *ampC* and *bla*_OXA-51-like_ alleles as of Ab-Pak-cluster-7. Both K50 and AA-014 belonged to ST158 [40, 41]. K50 was recovered from a hospitalized patient in Kuwait in 2008, while AA-014 was collected from a wound specimen in Iraq in 2008. Additional two isolates, 2226C and ABC002, were assigned into ST158 according to the MLST database (https://pubmlst.org/abaumannii/). 2226C was isolated in Turkey in 2009 while ABC002 was collected in Egypt in 2011 (https://pubmlst.org/). ST158 isolates were also reported in Lebanon between 2011 and 2013 [42]. The recognition of Ab-Pak-cluster-7 in Pakistan has then expanded the geographic distribution of ST158 beyond the Middle East [43–46].

### Antimicrobial resistance genes

A variety of antimicrobial resistance genes were detected in the genomes of the 25 isolates selected for whole genome sequencing (Additional file 7: Table S5). Class D ß-lactamase gene *bla*_OXA-23_ was present in 24/25 isolates. *bla*_OXA-23_ was located either in Tn*2006* (12 isolates) or Tn*2008* (12 isolates). Some isolates, such as Ab-Pak-Lah-04 and Ab-Pak-Lah-08 from Ab-Pak-cluster-1, carried three copies of *bla*_OXA-23_. Of note, only one copy was intact while the other two were truncated at their 5′ extremities (data not published). A similar scenario was detected in strain AbPK1 (GenBank: CP024576). Interestingly, AbPK1 was collected in Pakistan in 2012. It also belonged to ST2 and had the *ampC*-*19* and *bla*_OXA-66_ alleles [47]. Although it was isolated from infected animals, AbPK1 had a strong genotypic linkage to Ab-Pak-cluster-1.

The individual strain Ab-Pak-Pesh-22 was resistant to meropenem but susceptible to imipenem. Interestingly, Ab-Pak-Pesh-22 did not carry *bla*_OXA-23_ or any other commonly known mechanism for carbapenem resistance. On the other hand, Ab-Pak-Lah-01 was equipped with two mechanisms conferring carbapenem resistance, an acquired *bla*_OXA-23_ gene and an intrinsic *bla*_OXA-66_ gene proceeded by IS*Aba1* [48]. None of our isolates carried the class D ß-lactamase *bla*_OXA-24-like_ or *bla*_OXA-58-like_ genes.

Overall, our results were in line with previous studies nominating OXA-23 as the most commonly detected group of acquired Class D β-lactamases in *A. baumannii* both in the region and throughout the world [49–51]. The occurrence of IS*Aba1* upstream of *bla*_OXA-23_, *bla*_OXA-66_, *ampC*-*2*, *ampC*-*3*, *ampC*-*8*, *ampC*-*19*, *ampC*-*20*, *ampC*-*78*, or *ampC*-*79* has the potential to overexpress these genes, which might subsequently confer resistance to carbapenems (*bla*_OXA_) or ceftazidime (*ampC*) as previously proposed [48, 52, 53].

Class A extended-spectrum ß-lactamase genes were detected in 14/25 of the isolates, among which *bla*_PER-1_, *bla*_TEM-1D_, *bla*_GES-11_ and *bla*_PER-7_ were carried by 5, 5, 3, and 1 isolate, respectively. There was no co-existence of more than one class A ß-lactamase gene per isolate. All the isolates (25/25) carried aminoglycoside resistance genes. Each isolate carried between 2 to 7 aminoglycoside resistance genes. The occurrence rates ranged from 20/25 for *aphA6a* and *strB*, 16/25 for *strA*, 13/25 for *aacC1*, 8/25 for *aphA1b*, 7/25 for *aadA1*, 4/25 for *aphA6b*, *aadB*, and *aacA4*, and 1/25 for *aacC2*. The 16S rRNA methylase *armA* gene, commonly conferring high levels of resistance to aminoglycosides [54], was detected in 3 isolates, namely Ab-Pak-Pesh-04, Ab-Pak-Pesh-31, and Ab-Pak-Pesh-36. The inhibition zone diameters of gentamicin 10 μg, amikacin 30 μg, and tobramycin 10 μg for these *armA*-positive isolates did not show a unique resistance pattern compared to the *armA*-negative isolates (Additional file 3: Table S2).

The macrolide resistance 2′-phophotransferase *msr(E)*-*mph(E)* operon was present in 15/25 isolates. The sulphonamide resistance genes *sul2* and *sul1* were detected in 14/25 and 11/25 isolates, respectively, with 3 isolates having both *sul2* and *sul1*. The tetracycline resistance genes *tet(B)*, *tet(A)*, and *tet(39)* were detected in 9/25, 2/25, and 1/25 isolates, respectively, with no cases of overlapping. Other resistance genes, such as *floR*, *catA1*, *cmlA5*, and *catB8* (chloramphenicol resistance), *dfrA7* and *dfrA1* (trimethoprim resistance), and *arr*-*2* (rifampicin resistance) had low rates of occurrence ranging between 1/24 and 4/24 (Additional file 7: Table S5).

### Antimicrobial resistance features of Ab-Pak-cluster-7

**Fig. 2:**
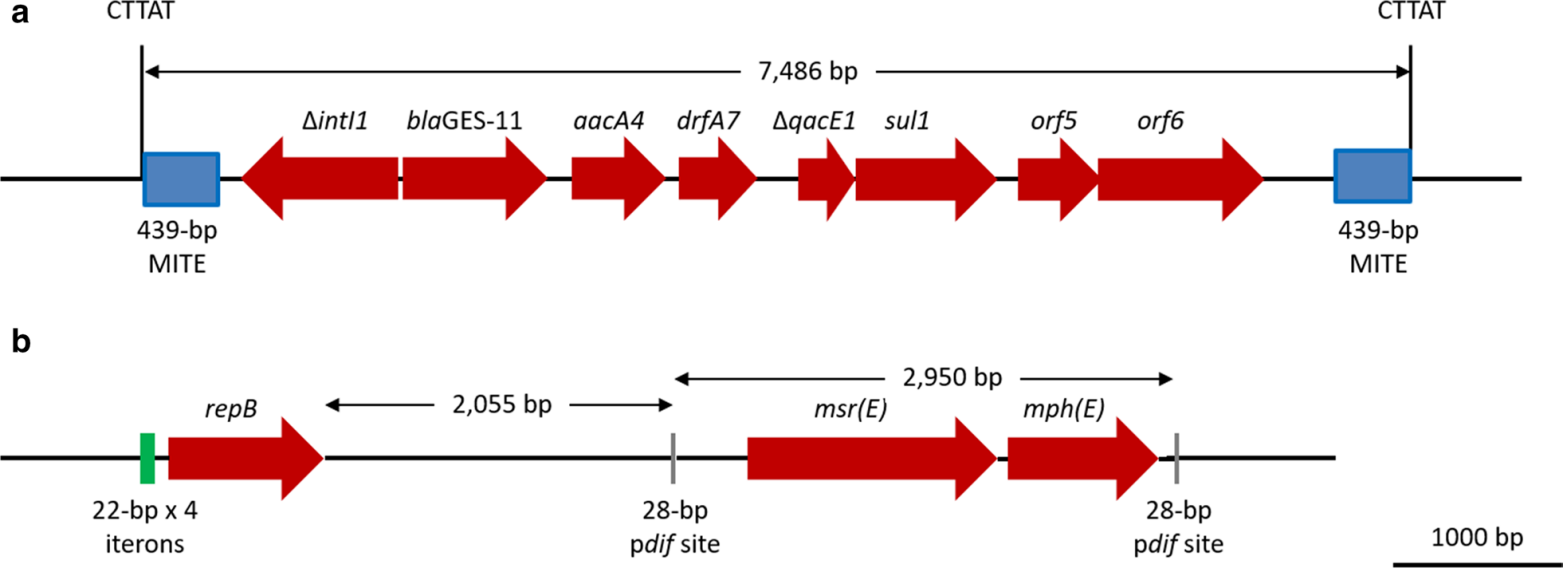
Genetic contexts of the *bla*_GES-11_ carbapenem resistance gene and the *msr(E)*-*mph(E)* macrolide resistance operon in Ab-Pak-cluster-7. **a***bla*_GES-11_ was located on a mobile element of 7486 bp bounded by two miniature inverted-repeat transposable elements (MITE). **b***msr(E)*-*mph(E)* was carried on a mobile module of 2950 bp surrounded with two p*dif* sites. Genes and open reading frames (*orf*) were demonstrated as labeled arrows with the arrowhead indicating the direction of transcription. The MITEs were shown as labeled blue boxes. The duplication of 5 base pairs was shown as adjacent vertical bars labelled with (CTTAT). The iterons’ region and p*dif* sites were shown as labeled green box and gray bars, respectively

Ab-Pak-cluster-7 was equipped with 8 acquired antimicrobial resistance genes, namely: *bla*_OXA-23_, *bla*_GES-11_, *aphA6a*, *aacA4*, *sul1*, *drfA7*, *msr(E)* and *mph(E)*. The *bla*_OXA-23_ gene was located on transposon Tn*2008* with genetic surroundings identical to plasmid pK50a carried by isolate K50 [41]. Four genes, *aacA4*, *drfA7*, *bla*_GES-11_ and *sul1*, were part of a truncated class one integron (Fig. 2). The integron was bounded by two miniature inverted-repeat transposable elements (MITE), forming a mobile element of 7486 bp. The insertion of this mobile element created a characteristic target site duplication of 5-bp [55]. The same element was previously found on plasmids pK50a [41], p1AB5075 (CP008707.1), and pAb8098 (KY022424.1). It was also detected in the whole genome sequence of isolates 428 (MDTS01000040.1) and IS-251 (AMEJ01000018.1). The *aphA6a* gene in Ab-Pak-cluster-7 was located on transposon Tn*aphA6*, inserted at the same location as previously described for plasmids pAb-G7-2 (KF669606.1) and pAbPK1b (CP024578.1). Of note, Tn*aphA6* was missing in isolate K50.

The *msr(E)*-*mph(E)* macrolide resistance operon in Ab-Pak-cluster-7 was surrounded by two p*dif* sites creating a module with a probable movability mediated by the XerC–XerD system [56]. The *msr(E)*-*mph(E)* operon was co-located on a contig carrying the *repAci25* gene, encoding for novel plasmid replication initiator protein from the Rep_3 protein family (pfam01051) and superfamily (cl19398). *repAci25* had a nucleotide identity of 91% to *repAci4* (GenBank: GU978998.1). A close allele to *repAci25*, with 99.6% nucleotide identity, was detected on plasmids p597A-14.8 (GenBank: CP033871.1), pAb825_36 (GenBank: MG100202.1), pAb244_7 (GenBank: MG520098.1), and pAb242_9 (GenBank: KY984045.1). Among other *Acinetobacter* species, *repAci25* was detected in the whole genome sequence of *Acinetobacter bereziniae* (100% nucleotide identity, GenBank: CDEL01000266.1) and *Acinetobacter radioresistens* (99.7% nucleotide identity, GenBank: PXJE01000090.1). Interestingly, plasmid pALWVS1.1 in *Acinetobacter lwoffii* strain VS15 had a gene showing 96.7% nucleotide identity to *repAci25* (GenBank: KX426232.1). The age of the permafrost sediment, from which VS15 was obtained, was estimated to be around 2 to 3 million years [57].

## Conclusion

Our study demonstrated that uncomplicated sequence typing schemes, based on comparative analysis of one or two intrinsic loci, such as *ampC* and *bla*_OXA-51-like_, could be a practical approach for rapid grouping of bacterial clinical isolates, such as *A. baumannii*. However, further validation of the results is commonly needed. The occurrence of a 100% rate of multidrug-resistant strains is alarming and worth a rapid action plan, including regular follow ups and urgent management procedures. The study enabled us to detect seven clusters of *A. baumannii* prevailing in two clinical settings in Pakistan. Two of these clusters, Ab-Pak-cluster-1 and -6, lasted for more than 2 years and were able to spread between Lahore and Peshawar. The predominance of IC II in Pakistan was in line with the intensive circulation of this clone worldwide (11). The frequent occurrence of isolates belonging to IC I underlined that this clone is still a key trouble-maker in several parts of the world, including Pakistan. Significantly, Pakistan and the Middle East could be a reservoir for under-detected clones of carbapenem-resistant *A. baumannii*, including the *bla*_OXA-23_- and *bla*_GES-11_-positive ST158. The clinical significance and virulence features of ST158, represented by Ab-Pak-cluster-7 in Pakistan, is worth further investigations.

## Supplementary information

**Additional file 1: Figure S1.** The geographic distance between the Combined Military Hospital in Lahore (CMH Lahore) and the Combined Military Hospital in Peshawar (CMH Peshawar) as drawn by Google Maps https://www.google.com/maps/dir/Combined+Military+Hospital,+Mall+Rd,+Peshawar+Cantonment,+Peshawar,+Khyber+Pakhtunkhwa,+Pakistan/CMH+Lahore,+Abdul+Rehman+Rd,+Saddar+Town,+Lahore,+Punjab,+Pakistan/@32.7700744,72.6522947,8z/data=!4m14!4m13!1m5!1m1!1s0x38d917bbe7a07855:0xf8ccf377d1a61673!2m2!1d71.5423502!2d34.0033769!1m5!1m1!1s0x39190517756f887b:0xc49fc1d23f55d0b0!2m2!1d74.3726762!2d31.5408931!3e0.

**Additional file 2: Table S1.** Primers used for amplification and sequencing of the *bla*_OXA-51-like_ and *ampC* loci in 52 clinical isolates of *Acinetobacter baumannii* collected in Pakistan between 2013 and 2015.

**Additional file 3: Table S2.** Antimicrobial susceptibility results for 52 clinical isolates of *Acinetobacter baumannii* collected in Pakistan between 2013 and 2015.

**Additional file 4: File S1.** CLUSTAL O(1.2.4) multiple sequence alignment of *ampC-3* and *ampC-8*.

**Additional file 5: Table S3.** Genome assembly features of 25 clinical isolates of *Acinetobacter baumannii* collected in Pakistan between 2013 and 2015.

**Additional file 6: Table S4.** Plasmid replicon genes in the whole genome sequences of 25 clinical isolates of *Acinetobacter baumannii* collected in Pakistan between 2013 and 2015.

**Additional file 7: Table S5.** Acquired antimicrobial resistance genes retrived from the whole genome sequences of 25 clinical isolates of *Acinetobacter baumannii* collected in Pakistan between 2013 and 2015.

## Data Availability

Not applicable.
