# Supplementary material for: Molecular epidemiology and antimicrobial resistance features of Acinetobacter baumannii clinical isolates from Pakistan
Source: Ann Clin Microbiol Antimicrob. 2020 Jan 15;19:2. doi: 10.1186/s12941-019-0344-7 (PMC6964048; doi:10.1186/s12941-019-0344-7)
Supplement: Supplementary file 1 — Additional file 1: Figure S1. The geographic distance between the Combined Military Hospital in Lahore (CMH Lahore) and the Combined Military Hospital in Peshawar (CMH Peshawar) as drawn by Google Maps https://www.google.com/maps/dir/Combined+Military+Hospital,+Mall+Rd,+Peshawar+Cantonment,+Peshawar,+Khyber+Pakhtunkhwa,+Pakistan/CMH+Lahore,+Abdul+Rehman+Rd,+Saddar+Town,+Lahore,+Punjab,+Pakistan/@32.7700744,72.6522947,8z/data=!4m14!4m13!1m5!1m1!1s0x38d917bbe7a07855:0xf8ccf377d1a61673!2m2!1d71.5423502!2d34.0033769!1m5!1m1!1s0x39190517756f887b:0xc49fc1d23f55d0b0!2m2!1d74.3726762!2d31.5408931!3e0. [file 12941_2019_344_MOESM1_ESM.docx]

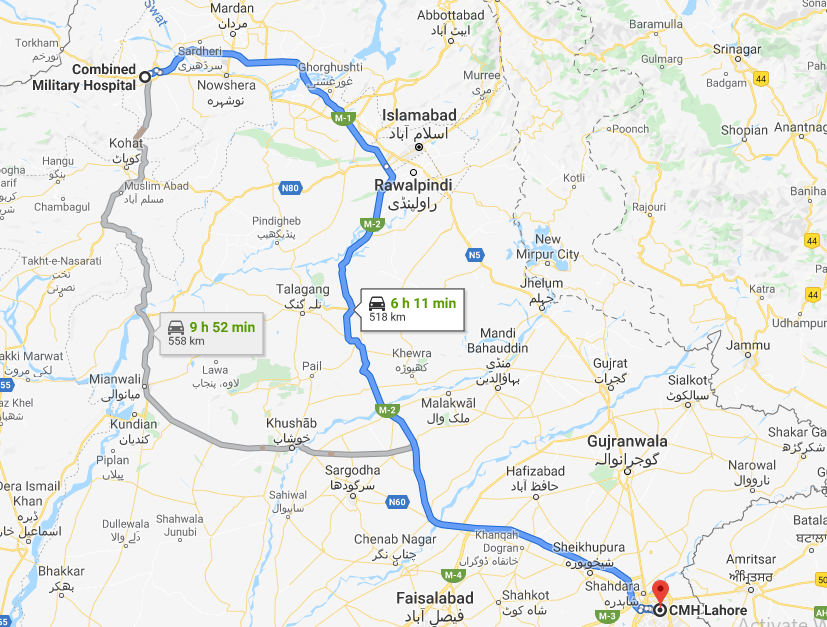


**Figure S1.** The geographic distance between the Combined Military Hospital in Lahore (CMH Lahore) and the Combined Military Hospital in Peshawar (CMH Peshawar) as drawn by Google Maps <https://www.google.com/maps/dir/Combined+Military+Hospital,+Mall+Rd,+Peshawar+Cantonment,+Peshawar,+Khyber+Pakhtunkhwa,+Pakistan/CMH+Lahore,+Abdul+Rehman+Rd,+Saddar+Town,+Lahore,+Punjab,+Pakistan/@32.7700744,72.6522947,8z/data=!4m14!4m13!1m5!1m1!1s0x38d917bbe7a07855:0xf8ccf377d1a61673!2m2!1d71.5423502!2d34.0033769!1m5!1m1!1s0x39190517756f887b:0xc49fc1d23f55d0b0!2m2!1d74.3726762!2d31.5408931!3e0>
