# Supplementary material for: Molecular epidemiology and antimicrobial resistance features of Acinetobacter baumannii clinical isolates from Pakistan
Source: Ann Clin Microbiol Antimicrob. 2020 Jan 15;19:2. doi: 10.1186/s12941-019-0344-7 (PMC6964048; doi:10.1186/s12941-019-0344-7)
Supplement: Supplementary file 4 — Additional file 4: File S1. CLUSTAL O(1.2.4) multiple sequence alignment of ampC-3 and ampC-8. [file 12941_2019_344_MOESM4_ESM.pdf]

CLUSTAL O(1.2.4) multiple sequence alignment of *ampC-3* and *ampC-8*

```
ampC-3      AGCTAATCATGCAATTTAAAAAAATTTCTTGTCTACTTTTATCCCCGCTTTTTATTTTTA 60
ampC-8      AGCTAATCATGCAATTTAAAAAAATTTCTTGTCTACTTTTATCCCCGCTTTTTATTTTTA 60
            ************************************************************

ampC-3      GTACCTCAATTTATGCGGACAATACACCAAAAGACCAAGAAATTAAAAAACTGGTAGATC 120
ampC-8      GTACCTCAATTTATGCGGACAATACACCAAAAGACCAAGAAATTAAAAAACTGGTAGATC 120
            ************************************************************

ampC-3      AAAATTTTAAACCATTATTAGAAAAATATGATGTGCCGGGTATGGCTGTGGGTGTTATTC 180
ampC-8      AAAATTTTAAACCATTATTAGAAAAATATGATGTGCCGGGTATGGCTGTGGGTGTTATTC 180
            ************************************************************

ampC-3      AAAATAATAAAAAGTATGAAATGTATTATGGTCTTCAATCTGTTCAAGATAAAAAAGCCG 240
ampC-8      AAAATAATAAAAAGTATGAAATGTATTATGGTCTTCAATCTGTTCAAGATAAAAAAGCCG 240
            ************************************************************

ampC-3      TAAATAGCAGTACTATTTTTGAGCTAGGTTCTGTCAGTAAATTATTTACCGCGACAGCAG 300
ampC-8      TAAATAGCAGTACTATTTTTGAGCTAGGTTCTGTCAGTAAATTATTTACCGCGACAGCAG 300
            ************************************************************

ampC-3      GTGGATATGCAAAAAATAAAGGAAAAATCTCTTTTGACGATACGCCTGGTAAATATTGGA 360
ampC-8      GTGGATATGCAAAAAATAAAGGAAAAATCTCTTTTGACGATACGCCTGGTAAATATTGGA 360
            ************************************************************

ampC-3      AAGAGCTAAAAAATACACCGATTGACCAAGTTAACTTACTTCAACTCGCGACGTATACAA 420
ampC-8      AAGAGCTAAAAAATACACCGATTGACCAAGTTAACTTACTTCAACTCGCGACGTATACAA 420
            ************************************************************

ampC-3      GTGGTAACCTTGCCTTGCAGTTCCCAGATGAAGTACAAACAGATCAACAAGTTTTAACTT 480
ampC-8      GTGGTAACCTTGCCTTGCAGTTCCCAGATGAAGTACAAACAGATCAACAAGTTTTAACTT 480
            ************************************************************

ampC-3      TTTTCAAAGACTGGAAACCTAAAAACCCAATCGGTGAATACAGACAATATTCAAATCCAA 540
ampC-8      TTTTCAAAGACTGGAAACCTAAAAACCCAATCGGTGAATACAGACAATATTCAAATCCAA 540
            ************************************************************

ampC-3      GTATTGGCCTATTTGGAAAGGTTGTAGCTTTGTCTATGAATAAACCTTTCGACCAAGTGT 600
ampC-8      GTATTGGCCTATTTGGAAAGGTTGTAGCTTTGTCTATGAATAAACCTTTCGACCAAGTGT 600
            ************************************************************

ampC-3      TAGAAAAAACAATTTTTCCGGCCCTTGGCTTAAAACATAGCTATGTAAATGTACCTAAGA 660
ampC-8      TAGAAAAAACAATTTTTCCGGCCCTTGGCTTAAAACATAGCTATGTAAATGTACCTAAGA 660
            ************************************************************

ampC-3      CCCAAATGCAAAACTATGCTTTTGGCTATAACCAAGAAAATCAGCCGATTCGAGTTAACC 720
ampC-8      CCCAAATGCAAAACTATGCTTTTGGCTATAACCAAGAAAATCAGCCGATTCGAGTTAACC 720
            ************************************************************

ampC-3      CCGGCCCACTCGATGCCCCAGCAT---------------ATGGCGTCAAATCGACACTAC 765
ampC-8      CCGGCCCACTCGATGCCCCAGCATATGGCGTCCCAGCATATGGCGTCAAATCGACACTAC 780
            ************************               *********************

ampC-3      CCGACATGTTGAGTTTTATTCATGCCAACCTTAACCCACAGAAATATCCGGCAGATATTC 825
ampC-8      CCGACATGTTGAGTTTTATTCATGCCAACCTTAACCCACAGAAATATCCGGCAGATATTC 840
            ************************************************************
```

```
ampC-3      AACGGGCAATTAATGAAACACATCAAGGGTTCTATCAAGTAAATACCATGTATCAGGCAC 885
ampC-8      AACGGGCAATTAATGAAACACATCAAGGGTTCTATCAAGTAAATACCATGTATCAGGCAC 900
            ************************************************************

ampC-3      TCGGTTGGGAAGAGTTTTCTTATCCGGCAACGTTACAAACTTTATTAGACAGTAATTCAG 945
ampC-8      TCGGTTGGGAAGAGTTTTCTTATCCGGCAACGTTACAAACTTTATTAGACAGTAATTCAG 960
            ************************************************************

ampC-3      AACAGATTGTGATGAAACCTAATAAAGTGACTGCTATTTCAAAGGAACCTTCAGTTAAGA 1005
ampC-8      AACAGATTGTGATGAAACCTAATAAAGTGACTGCTATTTCAAAGGAACCTTCAGTTAAGA 1020
            ************************************************************

ampC-3      TGTACCATAAAACTGGCTCAACTAACGGTTTCGGAACATATGTAGTGTTTATTCCTAAAG 1065
ampC-8      TGTACCATAAAACTGGCTCAACTAACGGTTTCGGAACATATGTAGTGTTTATTCCTAAAG 1080
            ************************************************************

ampC-3      AAAATATTGGTTTAGTCATGTTAACCAATAAACGTATTCCAAATGAAGAGCGCATTAAGG 1125
ampC-8      AAAATATTGGTTTAGTCATGTTAACCAATAAACGTATTCCAAATGAAGAGCGCATTAAGG 1140
            ************************************************************

ampC-3      CAGCTTATGCTGTGCTGAATGCAATAAAGAAATAA 1160
ampC-8      CAGCTTATGCTGTGCTGAATGCAATAAAGAAATAA 1175
            ***********************************
```
